# CenterPNets: A Multi-Task Shared Network for Traffic Perception

**DOI:** 10.3390/s23052467

**Published:** 2023-02-23

**Authors:** Guangqiu Chen, Tao Wu, Jin Duan, Qi Hu, Dandan Huang, Hao Li

**Affiliations:** 1 College of Electronic Information Engineering, Chang Chun University of Science and Technology, Changchun 130022, China; 2 College of Artificial Intelligence, Chang Chun University of Science and Technology, Changchun 130022, China

**Keywords:** traffic perception, multi-task learning, target detection, semantic segmentation

## Abstract

The importance of panoramic traffic perception tasks in autonomous driving is increasing, so shared networks with high accuracy are becoming increasingly important. In this paper, we propose a multi-task shared sensing network, called CenterPNets, that can perform the three major detection tasks of target detection, driving area segmentation, and lane detection in traffic sensing in one go and propose several key optimizations to improve the overall detection performance. First, this paper proposes an efficient detection head and segmentation head based on a shared path aggregation network to improve the overall reuse rate of CenterPNets and an efficient multi-task joint training loss function to optimize the model. Secondly, the detection head branch uses an anchor-free frame mechanism to automatically regress target location information to improve the inference speed of the model. Finally, the split-head branch fuses deep multi-scale features with shallow fine-grained features, ensuring that the extracted features are rich in detail. CenterPNets achieves an average detection accuracy of 75.8% on the publicly available large-scale Berkeley DeepDrive dataset, with an intersection ratio of 92.8% and 32.1% for driveableareas and lane areas, respectively. Therefore, CenterPNets is a precise and effective solution to the multi-tasking detection issue.

## 1. Introduction

In recent years, the rapid development of embedded systems and neural networks has made autonomous driving a popular field in computer vision, where panoramic traffic perception systems play a crucial role in autonomous driving. Research has shown that vehicle onboard camera image processing enables scene understanding, including road target detection, driveable area detection, and lane detection, which greatly reduces overhead compared to the traditional approach of using LIDAR and millimeter wave radar to establish the vehicle’s surroundings.

The traffic panorama perception system’s detection precision and decision-making speed significantly influence the vehicle’s judgment and decision-making and determine the safety of autonomous vehicles. However, actual vehicle driver assistance systems, such asthe Advanced Driver Assistance System, have limited computing power and are expensive. Therefore, achieving a good balance between detection accuracy and model complexity from a practical application perspective is a challenge for decision-makers.

Current target detection can be broadly divided into one-stage detection models and two-stage detection models. The two-stage detection approach usually starts by acquiring a candidate region and then performing a regression prediction from that candidate region to ensure the accuracy of the detection. However, this step-by-step detection approach is not friendly to embedded systems. The end-to-end, one-stage detection model has the advantage of fast inference speed and is gaining more attention in the field of detection.The use of direct regression bounding boxes in the SSD [1] series, YOLO [2] series, etc. is a milestone in the first stage of detection. FCN [3] was the first to introduce fully convolutional networks to the task of semantic segmentation, although their performance was limited by resolution. PSPNet [4] proposes pyramid pooling to extract multi-scale features to improve detection performance. Enet [5] reduces the size of the feature map. SSN [6] incorporates conditional random field units in the post-processing stage to improve segmentation performance. LaneNet [7] proposes to use a single lane line as the object of instance segmentation. Layer-by-layer convolution is a technique used by spatial CNN [8] that enables the transfer of feature information between ranks in a layer. Enet SAD [9], on the other hand, uses a self-focused distillation method so that the feature maps can learn from each other.

Although the above algorithms are effective for their respective single-task detections, they can cause unnecessary network delays if they are used to achieve multi-task detection by acquiring the corresponding features through different task networks one by one. Multi-tasking networks, however, are better achieved by sharing information between multiple tasks. Mask R-CNN [10] extends Faster R-CNN [11] by mask branching to parallelize the detection task with the segmentation task. A similar approach is used by LSNet [12] for tasks such astarget detection, instance segmentation, etc. An encoder–decoder structure is suggested by MultiNet [13] to carry out the scene perception job concurrently.On the BDD100K dataset [14], YOLOP [15] is the first multi-tasking problem that implements panoramic driving perception, i.e., traffic target detection, driveable area segmentation, and lane detection, with high accuracy and speed at the same time with the help of embedded devices. YOLOP uses an efficient network structure and passes the feature information extracted from the images to the different decoders for their respective detection tasks. However, the use of separate segmentation heads for the driveable area and lane line segmentation tasks leaves room for multi-network optimization, i.e., these tasks can be fused into an overall segmentation task. HybridNets [16] uses a lighter backbone network than YOLOP, and to achieve a higher level of feature fusion, the neck network uses a weighted bi-directional feature network that treats each top-down and bottom-up bi-directional path as a feature network layer. However, the anchor mechanism is used in the detection task to return vehicle position information, which requires pre-clustering of anchor boxes in order to better fit the target size and has cumbersome subsequent processing during the prediction process. In this paper, we propose a more efficient multi-tasking network after a thorough study of previous approaches and incorporating the idea of an anchor-free architecture.

The CenterPNets backbone feature extraction network uses the CSPDarknet [17] module pre-trained on ImageNet and fuses the path aggregation neck network to achieve a good balance between detection accuracy and computational overhead. The PANet [18] decoder uses multi-scale feature data for tasks such as segmentation and detection. For model optimization, CenterPNets employs a multi-task joint loss function. CenterPNets abandons the anchor mechanism with high recall in the detection head in favor of an anchor-free mechanism that returns the target center position information without the need for time-consuming anchor frame clustering and subsequent processing, such as NMS, thereby increasing the network’s overall inference speed. In the segmentation task, shallow features are rich in fine-grained information, which is essential for image segmentation. For this reason, we fuse multi-scale features with shallow features to retain the detailed information of the image and make the segmented edges smoother.

In this paper, in addition to using an end-to-end training strategy, we have also tried a frozen training approach. Using the freeze training strategy, this approach has been shown to be effective at preventing information interference from other non-relevant modules in the network and the completed training tasks are instructive for other tasks.

To sum up, the main contributions of this research are: (1) This paper proposes an effective end-to-end shared multi-task network structure that can jointly handle three important traffic sensing tasks: lane detection, driveable area segmentation, and road target detection. The network’s encoders and decoders are shared to fully exploit the correlation between each task’s semantic features, which can help the network reduce model redundancy. (2) The detection head adopts an anchor-free mechanism to directly return the target key point information, size, and offset, without the need for pre-clustering anchor box ratio and tedious subsequent processing, thus enhancing the overall inference speed of the network. (3) In the segmentation head section, similar features from the shared detection task are used and proposed to fuse multi-scale, deep semantic information with shallow features so that the feature information extracted from the segmentation task is rich in fine-grained information, thus enhancing the detail segmentation capability of the model.

## 2. Methods

### 2.1. Network Architecture

**Figure 1:**
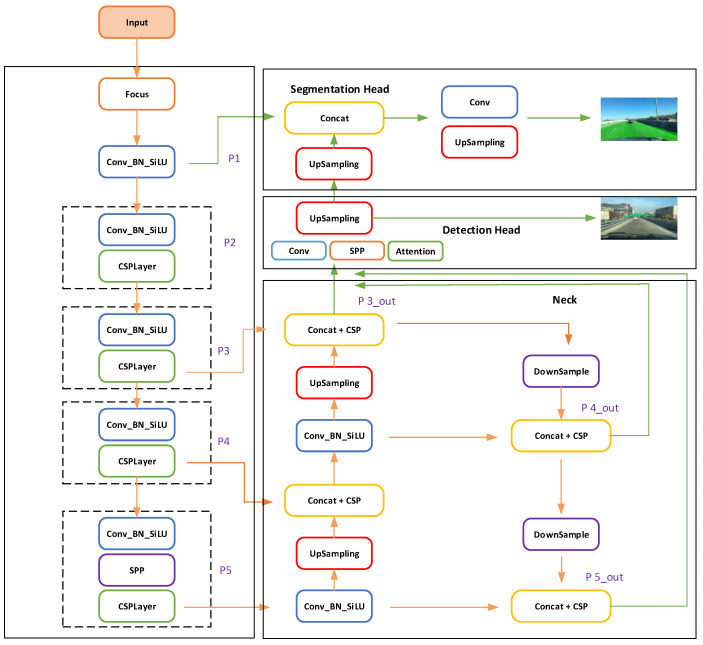
HybridNets Architecture has one encoder: backbone network and neck network;two decoders: Detection Head and Segmentation Head.

This paper proposes a multi-task traffic panorama perception architecture that can be jointly trained, called CenterPNets. As shown in Figure 1, the structure mainly contains encoders, decoders, and task-independent detection heads to handle the corresponding detection tasks, and there are no redundant parts between the modules, which reduces computational consumption to a certain extent.

In the encoder part, feature extraction is the core structure in the network, which directly determines the accuracy of the network detection. Many modern networks currently extract features directly using networks that have good detection performance in the ImageNet dataset. One of the most traditional deep networks, Darknet, combines Resnet [19] features to ensure excellent feature representation while avoiding the gradient issues that come with overlying deep networks. CenterPNetsusesCSPDarkNet as the backbone, which combines the advantages of CSPNet and SPP [20] modules to maximize the difference in gradient union, and its use of gradient stream splitting and merging to avoid different layers of learning to duplicate gradient information is effective enough to reduce duplicate gradient learning. As a result, the backbone network of CenterPNets can extract crucial feature information while lowering the network’s computational cost.

The feature map extracted by the encoder is passed to the neck structure of the network. The Feature Pyramid Network(FPN) module [21] a feature extractor design for generating multi-scale feature maps to obtain better information. However, the limitation of FPN is that the information features are inherited by a uni-directional flow. As a result, the CenterPNets neck network makes use of the PANet module with the addition of a top-down feature pyramid behind the FPN layer. Through its structural properties, it can effectively compensate for the fact that FPN only enhances the semantic information of the feature pyramid and lacks localization information.

A. Anchor-free detection head

**Figure 2:**
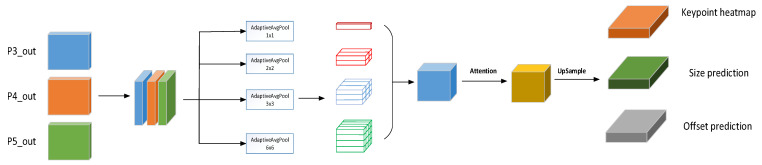
Illustration of the detection head branching process.

As shown in Figure 2, in the detection head section, CenterPNets integrates information from the P3_out, P4_out, and P5_out multi-level feature maps in the neck network at the same resolution in order to obtain multi-level semantic features, followed by pyramid pooling and attention mechanisms to reinforce the relevant feature information, which is recovered by upsampling to a feature map with 1/4 of the input image resolution. CenterPNets uses an anchor-free mechanism [22] for direct regression prediction, eliminating the need for K-means clustering to determine pre-defined anchor box proportions and tedious NMS follow-up, allowing for direct regression of key point heatmaps, size prediction, and offset prediction, thus improving the overall speed of inference in the network.

Keypoint heatmap:

Assume that the input image is $I\in R^{W•H•3}$, where *W* and *H* are the width and height of the input image, respectively, C is the category type of the detection target, and *R* is the output step. In this paper, only the car category label is detected, so *C* = 1. We use the default output step of *R* = 4 and deflate the output prediction by *R*. For the real category labeling point $P\in R^{2}$, a low-resolution equivalent point $\hat{P}=\left\lfloor \frac{P}{R}\right\rfloor$ is used instead. Generate a heat map of key points $\hat{Y}\in {\left[0,1\right]}^{W\times H\times C}$ during model training, where C represents the number of category labels detected.In this paper, only the car category is detected. $\hat{Y}=1$ means that the target to be measured is detected at (*x*, *y*) out.$\hat{Y}=0$ indicates a background area. For each ground truth keypoint *P*, we splat it onto a heatmap using a Gaussian kernel $Y_{\mathrm{xyc}}=\exp\left(-\frac{{\left(x-p_{x}\right)}^{2}+{\left(y-p_{y}\right)}^{2}}{2\delta_{p}^{2}}\right)$, $\delta_{p}$ is an object size-adaptive standard deviation [23]. When there are two Gaussian kernels that overlap, the maximum value of the elements is taken in this paper [24]. The difference between the predicted and real heatmaps is the pixel-wise focus loss [25].

(1) $$L_{k}=-\frac{1}{N}\sum_{xyc}\{\begin{array}{cc} {\left(1-{\hat{Y}}_{xyc}\right)}^{\alpha }\log({\hat{Y}}_{xyc}) & Y_{xyc}=1\\ {\left(1-Y_{xyc}\right)}^{\beta }{\left({\hat{Y}}_{xyc}\right)}^{\alpha } & otherwise\\ \log(1-{\hat{Y}}_{xyc}) & otherwise \end{array}\;$$

where *α* and *β* are hyperparameters of focal loss and *N* is the number of critical points. In our experiments, we used *α* = 2, *β* = 4 [23].

Size prediction:

Assume that the kth bounding box has coordinates $\left(x_{1}^{k},y_{1}^{k},x_{2}^{k},y_{2}^{k}\right)$ and a width and height $s_{k}=\left(x_{2}^{k}-x_{1}^{k},y_{2}^{k}-y_{1}^{k}\right)$. The coordinates of its center point are $p_{k}=\left(\frac{x_{1}^{k}+x_{2}^{k}}{2},\frac{y_{1}^{k}+y_{2}^{k}}{2}\right)$. We calculate the predicted loss using *L*_1_ only at the center of the target.

(2) $$L_{size}=\frac{1}{N}\sum_{k=1}^{N}\left|{\hat{S}}_{p_{k}}-s_{k}\right|$$

Offset prediction:

The output feature map will contain accuracy errors when remapping to the original image size because the decoder outputs features at a resolution that is one-fourth that of the original input image. As a result, an extra local offset is applied for each key point to make up for the inaccuracy.

(3) $$L_{off}=\frac{1}{N}\sum_{p}\left|{\hat{O}}_{\tilde{p}}-\left(\frac{p}{R}-\tilde{p}\right)\right|$$

where ${\tilde{O}}_{\;\tilde{p}}$ denotes the offset of the network prediction, *P* denotes the image centroid coordinates, and R denotes the heatmap scaling factor.

B. Segmentation heads incorporating fine-grained features

**Figure 3:**
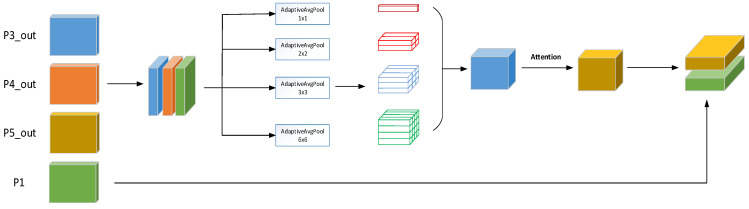
Illustration of the branching process of the segmented head.

As shown in Figure 3, the segmented head section outputs 3 categories of labels, namely background, road trafficable area, and road lane lines. There is a correlation between the feature information of the detection task and the segmentation task, so CenterPNets shares the same feature mapping between the two and upsamples the feature fusion with the shallow, fine-grained feature P1 layer with rich localization information based on the detection feature mapping, thus enhancing the network’s ability to segment image edge details. Finally, we recover the output features to the original image resolution (W, H, 3), storing the probability values for each pixel category label.

### 2.2. Loss of Function for Joint Multi-Task Training

The end-to-end network is trained by CenterPNets using a multi-task loss function, which sums two components to represent the entire loss function.

(4) $$L_{\mathrm{all}}=\alpha L_{\det}+\beta L_{seg}$$

where *L*_det_ is the target detection loss and *L_seg_* is the semantic segmentation loss. *α*, *β* are the balance factors of the loss function in order to keep the detection task in the same order of magnitude as the segmentation task.

(5) $$L_{\det}=L_{k}+\lambda_{size}L_{size}+\lambda_{off}L_{off}$$

where $L_{size}$, $L_{\mathrm{off}}$ use the ordinary *L*_1_ loss function, which is used to regress the width and height and centroid offsets, respectively. For heat map losses, $L_{k}$ is calculated by focal loss.

(6) $$L_{k}=-\frac{1}{N}\sum_{xyc}\{\begin{array}{cc} {{(1-\;\hat{Y}}_{\mathrm{xyc}})}^{\alpha }\log\left({\hat{Y}}_{xyc}\right) & Y_{xyc}=1\\ {\left(1-Y_{xyc}\right)}^{\beta }{\left({\hat{Y}}_{xyc}\right)}^{\alpha } & otherwise \end{array}$$

Multi-class mixture loss is used for multi-class segmentation of backgrounds, driveable areas, and lane lines. Semantic segmentation is difficult due to the uneven distribution of data. Therefore, CenterPNets combines Tversky loss $L_{Tversky}$ [26] and focus loss $L_{F\mathrm{ocal}}$ [27] to predict the class to which the pixel belongs. $L_{Tversky}$ performs well on the class imbalance problem and is optimized for score maximization, while $L_{F\mathrm{ocal}}$ aims to minimize classification errors between pixels and focuses on hard labeling.

(7) $$L_{\mathrm{seg}}=L_{Tversky}+L_{Focal}$$

(8) $$L_{Tversky}=C-\sum_{C=0}^{C-1}\frac{TP_{p}(c)}{TP_{p}(c)+\varphi FN_{p}(c)+(1-\varphi )FP_{p}(c)}$$

(9) $$L_{F\mathrm{ocal}}=-\lambda \frac{1}{N}\sum_{c=0}^{C-1}\sum_{n=1}^{N}g_{n}(c){\left(1-p_{n}(c)\right)}^{\gamma }\log(p_{n}(c))$$

where $TP_{p}\left(c\right)$, $FN_{p}\left(c\right)$, and $FP_{p}\left(c\right)$ are classes of true positives, false negatives, and false positives. $P_{n}\left(c\right)$ is the predicted probability of a class of pixels. $g_{n}(c)$ is denoted as the true annotation category C for pixel n. *C* is the number of classes in Equation (8) and *N* is the total number of pixels in the input image in Equation (9).

## 3. Results

### 3.1. Setting

#### 3.1.1. Dataset Setting

The experiments in this paper use image data from the Berkeley DeepDrive dataset (BDD100K) to train and validate the model. Existing multi-task networks are trained against datasets from three tasks on BDD100K to help compare performance with other models. In the target detection task, “car, truck, bus, train” are combined into a single category label “car,” as MultiNet, YOLOP, and HybridNets can only detect vehicle category labels. Basic enhancements such as rotation, scaling etc. are used in image pre-processing.

#### 3.1.2. Implementation Details

This studyperforms backbone initialization by using CSPDarkNet weights pre-trained on ImageNets. The optimizer uses AdamW [28], where $\gamma =1\times 10^{-3},\beta_{1}=0.9,\beta_{2}=0.999,\xi =1\times 10^{-8},\lambda =1\times 10^{-2}$. The learning rate is a non-linear approach with its initial value set to $1\times 10^{-5}$. Optimization uses *L*_1_ and focal loss in the target detection task, where $\lambda_{size}=0.1,\lambda_{off}=1$. For the driveable area and lane splitting, the model uses a combination of Tversky loss and focal loss. In this study, 200 cycles are trained on RTXA4000.

#### 3.1.3. Evaluation Indicators

In the traffic target detection task, performance is evaluated with the help of mAP50. mAP50 is calculated by averaging the average accuracy of the categories below a single IoU threshold of 0.5.

(10) $$AP=\sum_{i=1}^{n-1}\left(r_{i+1}-r_{i})P_{\mathrm{int}er}(r_{i}+1\right)$$

where *r*_1_, *r*_2_, …, *r_n_* are the recall values corresponding to the first interpolation of the precision interpolation segment in ascending order.

(11) $$mAP=\frac{\sum_{i=1}^{k}AP_{i}}{k}\;$$

In the semantic segmentation task, the IoU metric is used to evaluate the driveable area and lane line segmentation. In this paper, mIoU is represented as the average IoU per class and the IoU metric for individual classes. In order to illustrate the validity of the experiment more favorably, accuracy has been added as an additional criterion.

(12) $$IoU=\frac{B_{t}\wedge B_{p}}{B_{t}\cup B_{p}}$$

where *B_p_* is the predicted bounding box and $B_{t}$ is ground truth bounding box.

### 3.2. Experimental Analysis of Multi-Tasking Networks

In this section, we first train the model end-to-end, then compare it with other representative models in the corresponding tasks and illustrate the effect of each module on the network and the effectiveness of multi-task network learning by means of ablation experiments and freeze-out training, respectively.

#### 3.2.1. Road Target Detection Tasks

The CenterPNets algorithm istestedfor vehicle target detection on the BDD100K dataset and the algorithms are compared with MultiNet, Faster R-CNN, YOLOP, and HybridNets, and their experimental results are shown in Table 1.

As shown in Table 1, CenterPNets uses detection accuracy (mAP50) and recall (recall) as evaluation metrics. The CenterPNets model outperforms MultiNet and Faster R-CNN networks in terms of detection accuracy, but falls short of YOLOP and HybridNets. Since YOLOP uses a network structure based on the anchor box mechanism of YOLOV4, it has a high recall rate in feature regression by generating a dense anchor box approach, which allows the network to perform target classification and bounding box coordinate regression directly on this basis; HybridNets also uses a similar mechanism. CenterPNets, on the other hand, uses an anchor-free box mechanism, which results in average regression box quality because the anchor-free mechanism only predicts at locations closer to the center of the real box. As a result, it underperforms in terms of performance metrics when compared to YOLOP and HybridNets.

In this study, we use the same image and video data to verify the inference speed of the model. As can be seen from Table 2, the number of model parameters in this study has increased compared to the benchmark algorithm, which is due to the deeper network structure we have used to ensure detection performance. Secondly, the overall structure of the network in this studyis more integrated and eliminates tedious subsequent processing, etc., which optimizes the network to a certain extent. As can be seen from Table 2, the CenterPNets algorithm has an inference speed of 8.709 FPS when we perform unified image inference, compared to 5.719 FPS for HybridNets network inference, which shows a roughly 1.5-times improvement in inference speed, as the anchorless framework mechanism eliminates tedious subsequent processing. To further verify the reliability of the experiments, CenterPNetswastested uniformly using video data for inference, and it can be seen that the inference performance of CenterPNets is still very good.

**Figure 4:**
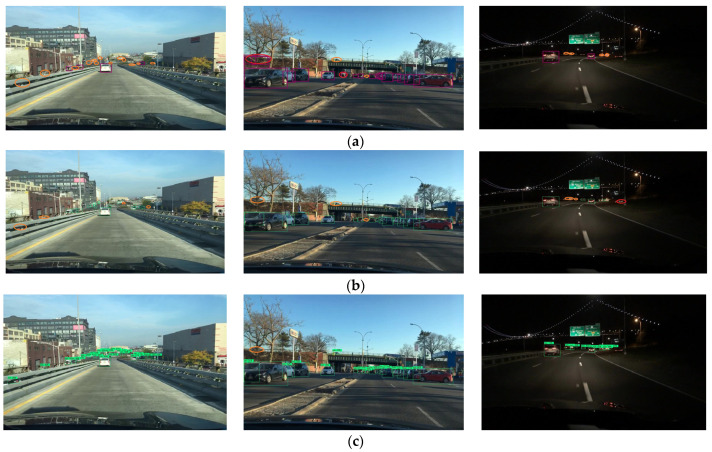
Target detection visualization comparison results. (**a**) YOLOP, (**b**) HybridNets, (**c**) CenterPNets.

To further evaluate the effectiveness of CenterPNets in real road traffic scenarios, images of road scenes at different times of the day areselected from the BDD100K test set for experimental effectiveness testing. The YOLOP, HybridNets, and CenterPNets algorithms for traffic target recognition tasks at various times of the day are visually compared in Figure 4. The first row displays the results of the YOLOP test, the second row the results of the HybridNets test, and the third row the results of the CenterPNets test. Orange circles denote false negatives, and red circles false positives. The CenterPNets shared network architecture is a further improvement compared to YOLOP and HybridNets. As shown, it can be seen that YOLOP and HybridNets both have a certain degree of missed and false vehicle target detection, while the CenterPNets algorithm has better vehicle target detection capabilities and more accurate bounding boxes in different environments.

#### 3.2.2. Driveable Area and Lane Detection Tasks

A. Travelable area segmentation tasks

CenterPNets uses the IoU metric to evaluate the driveable area segmentation capability and is compared with the algorithms MultiNet, PSPNet, YOLOP, and HybridNets, whose experimental results are shown in Table 3.

The driveable portion of the image and the backdrop are the only things the CenterPNets model needs to differentiate between. Comparing the five driveable area detection networks, Table 3 demonstrates that the CenterPNets algorithm had the highest mIoU performance of 92.8%, an improvement of 1.3% and 2.3% over YOLOP and HybridNets, respectively. Due to the feature correlation between the road vehicle detection task and the road travel area segmentation task, the CenterPNets shared network can effectively information correlation between the two; secondly, CenterPNets first fuses deep multi-scale features and combines shallow feature information so that the extracted semantic feature information has local fine-grained information at the same time, smoothing the road edge segmentation.

**Figure 5:**
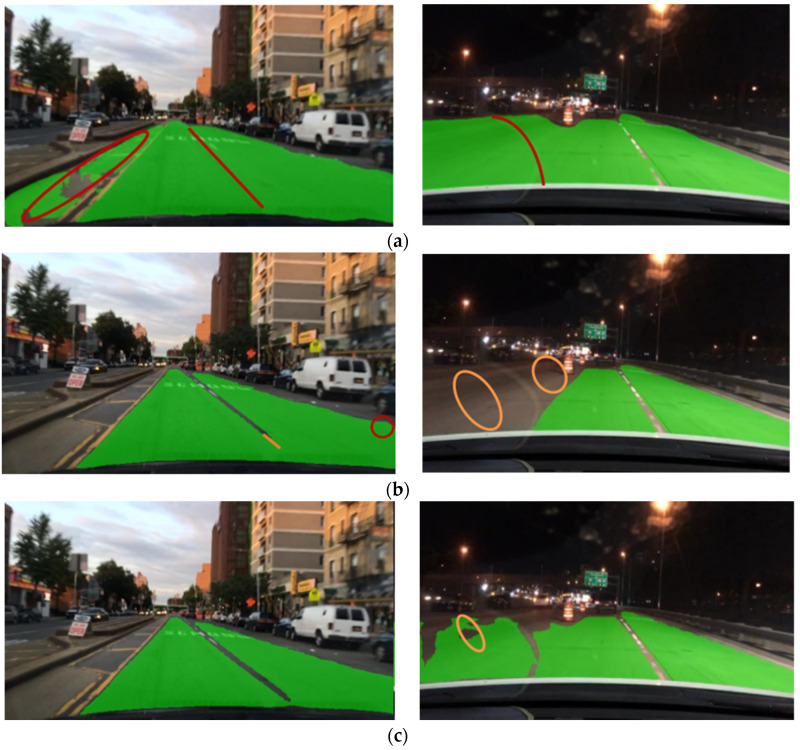
Comparative results of the visualization of the driveable area segmentation. (**a**) YOLOP, (**b**) HybridNets, (**c**) CenterPNets.

For the driveable area segmentation task, in Figure 5, red in the depiction is a false positive and orange is a false negative. The CenterPNets method is more precise than YOLOP and HybridNets region segmentation, as demonstrated by a visual comparison of the CenterPNets network with those two algorithms. YOLOP considers the intersection of bounding boxes while concentrating on determining the class to which the pixel belongs. As a result, the YOLOP model’s detection suffers from some lane line and road area misdetection as well as an inability to precisely segment the driveable portion of the road. For the neck network, HybridNets uses a BiFPN architecture, in which information from different receptive fields is combined from different feature map levels by weighting parameters, an improvement over the YOLOP segmentation structure but still with regional underdetection. The CenterPNets algorithm uses the PANet architecture of the neck network to fuse different scale features to make the global information richer, while taking advantage of the correlation between multi-task features and combining it with rich shallow fine-grained feature information to ensure that the network captures more detailed information. The driveable area segmentation task can therefore be effectively improved by the CenterPNets network. The CenterPNets method exhibits some inadequate area segmentation at complicated junctions, as seen in the picture, but the overall highway driveable area may be more precisely segregated from the backdrop and lane lines.

B. Lane area splitting task

Lane detection is one of the main challenges for autonomous driving. CenterPNets uses accuracy and IoU as evaluation metrics for lane detection and compares the algorithms with ENet, SCNN, YOLOP, and HybridNets, whose experimental results are shown in Table 4.

As shown in Table 4, CenterPNets’ shared network multi-tasking architecture accomplished both the driving area and lane line segmentation tasks in the segmentation head section, with the CenterPNets algorithm achieving the best performance results of 86.20% accuracy and 32.1% IoU, an improvement in performance compared to other detection networks.

**Figure 6:**
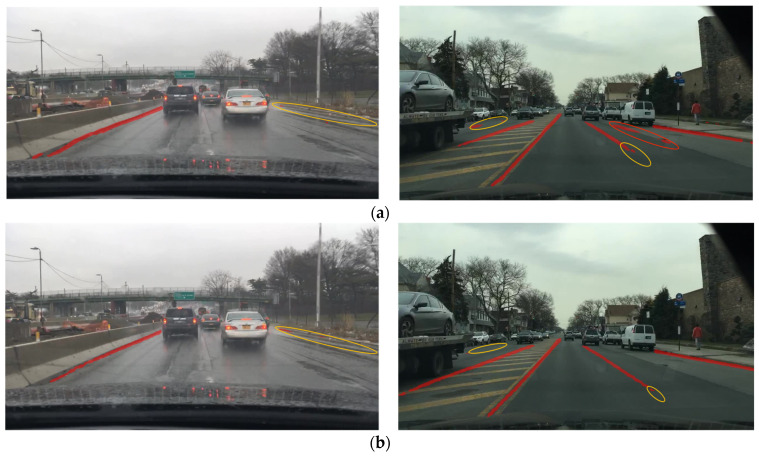

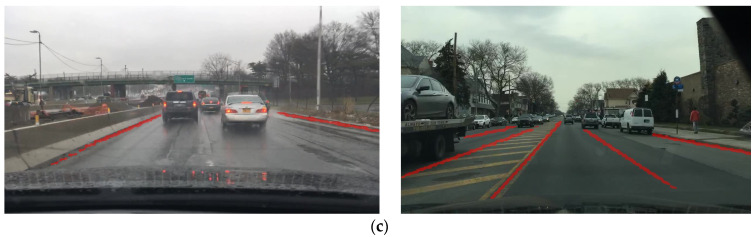
Comparison of lane split visualization results. (**a**) YOLOP, (**b**) HybridNets, (**c**) CenterPNets.

As shown in Figure 6 on the lane segmentation task, with the orange circles showing false negatives and the red circles false positives, a visual comparison of the CenterPNets network with YOLOP and HybridNet shows that there is a degree of lane pixel underdetection in YOLOP and HybridNets, while the features extracted by the CenterPNets algorithm have richer global fine-grained information. As a result, it excels in lane detection, and the outcomes of lane segmentation are more continuous and complete.

C. Split task ablation experiment

CenterPNets is further used to analyze the impact of modules such as multi-scale feature information (MFI),spatial pyramidal pooling (SPP), attention mechanism (Attention), and superficial feature information (SCI) on the segmentation task. As can be seen from Experiments 1 and 2 in Table 5, by introducing multi-scale information fusion, the driveable area IoU and accuracy improved by 3.8% and 1.9%, respectively, and the lane detection IoU and accuracy improved by 2.8% and 2.3%, respectively, thus demonstrating the effectiveness of multi-level contextual feature information for the segmentation task. Experiments 2, 3, 4, and 5 show that the spatial pyramid pooling and attention mechanism effectively enhance the road-related area features, with 1.0% and 1.8% improvements in the IoU and accuracy of the lane lines, respectively.

#### 3.2.3. Training Method Comparison Experiment

In order to verify the effectiveness of joint multi-task training, this paper compares the impact of the multi-task training approach and the single-task training approach on the overall performance of the network. Table 6 shows a comparison of the performance of these two schemes on their specific tasks. It can be seen that the overall performance of the model in this paper using a multi-task training scheme outperforms the performance of the individual tasks. More importantly, the multi-task model can save a significant amount of inference time compared to performing the respective tasks individually.

**Figure 7:**
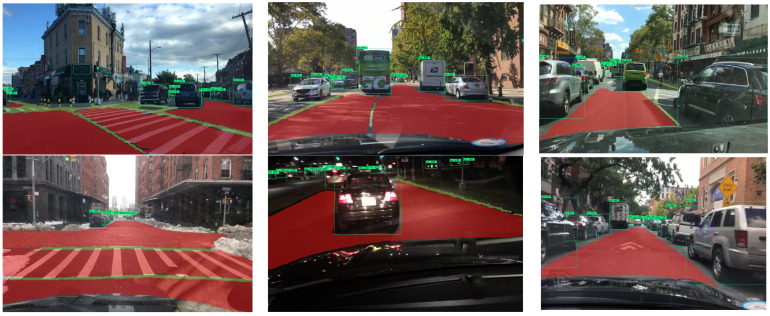
CenterPNets multi-tasking results.

Figure 7 shows the results of some of the CenterPNets tests, where yellow is the lane line, red is the driveable area, and the green border is the traffic vehicle target. As can be seen, CenterPNets performed relatively well in most cases.CenterPNets exploits the correlation between the detection task and the segmentation task based on contextual information in order to help the training model converge more quickly. Therefore, CenterPNets in this paper can perform the traffic perception task more easily. In general, CenterPNets can perform the detection task well in the vast majority of scenarios. However, there are still some lane prediction interruptions and missed detections at complex intersections.

## 4. Conclusions

In this paper, the effectiveness of multi-task network detection is systematically described, and a perceptual structure called theCenterPNets shared codec is proposed to integrate multi-scale feature information through a path aggregation network, which is used for direct regression to target key points.In the semantic segmentation task, the detailed information of the image is enhanced by fusing the multi-level features of the path aggregation network with shallow fine-grained information and building an effective training loss function to improve accuracy and performance.CenterPNets achieved an average detection accuracy of 75.8% on the publicly available large-scale Berkeley DeepDrive dataset, with an average intersection ratio of 92.8% in the driveable area and 32.1% in the lane area, respectively. Compared to the baseline algorithm, CenterPNets showed a 2.3% and 0.5% improvement in the cross-merge ratio for the roadway driveable area and lane line segmentation tasks, respectively. More importantly, CenterPNets achieved more accurate traffic segmentation tasks with relatively fast inference compared to other multi-task detection networks.

## Figures and Tables

**Table 1:** Comparison results of road target detection algorithms.

Model	Recall (%)	mAP50 (%)
MultiNet	81.3	60.2
Faster R-CNN	77.2	55.6
YOLOP	89.2	76.5
HybridNets	92.8	77.3
CenterPNets	81.6	75.8

**Table 2:** Inference speed for vehicle target detection.

Model	Image_infer(FPS)	Video_infer(S)	Param(M)
HybridNets	5.719	90.632	12.83
CenterPNets	8.709	68.279	28.56

**Table 3:** Performance comparison for the driveable area segmentation task.

Model	DriveablemIoU (%)
MultiNet	71.6
PSPNet	89.6
YOLOP	91.5
HybridNets	90.5
CenterPNets	92.8

**Table 4:** Performance comparison of lane detection tasks.

Model	Accuracy (%)	Lane Line IoU (%)
ENet	34.12	14.64
SCNN	35.79	15.84
YOLOP	70.50	26.20
HybridNets	85.40	31.60
CenterPNets	86.20	32.10

**Table 5:** Experimental analysis of semantic segmentation task ablation.

Experimental Serial Number	MFI	SPP	Attention	SCI	Driveable		Lane Line	
					IoU (%)	Acc (%)	IoU (%)	Acc (%)
1	---	---	---	---	78.7	93.2	28.3	81.2
2	√	---	---	---	82.5	95.1	31.1	83.5
3	√	√	---	---	82.2	94.6	31.3	84.8
4	√	√	√	---	82.9	94.8	31.5	85.5
5	√	√	√	√	82.6	94.2	32.1	85.3

**Table 6:** The experimental results of various training modalities.

Training Method	Detection		Driveable		Lane Line	
	Recall (%)	AP (%)	IoU (%)	Acc (%)	IoU (%)	Acc (%)
Only(det)	80.4	74.2	--	--	--	--
Only(seg)	--	--	82.6	94.2	32.1	85.3
Multi-task	81.6	75.8	82.5	93.2	32.1	86.2

## Data Availability

Not applicable.
